# The Week in the Courts

**Published:** 1911-03-04

**Authors:** 


### THE WEEK IN THE COURTS

### ACTION AGAINST A BONESETTER.

The hearing of the action against Mr. H. A. Barker, bonesetter, of Park Lane, which was mentioned in The Hospital last week, was concluded on February 22. The plaintiff, Mr. C. R. Thomas, sued Mr. Barker for damages for alleged negligence in the performance of operations which were alleged to have resulted in the amputation of the plaintiff's leg. The plaintiff's case was that while at Shrewsbury School in 1901 he twisted his knee in diving. From that time onward he suffered weakness and stiffness of the knee from time to time. Between 1906 and 1908 the knee gave him serious trouble, and after having been under treatment by medical practitioners he went to see Mr. Barker. He was put under an anæsthetic and, so it was alleged, was subjected on two occasions to violent treatment to "break down the adhesions." About a fortnight later a sore appeared on the side of the leg and it was contended that this was caused by bacilli which had been set free from the knee by the violent twists given on the occasions of the operations. The knee was in fact tuberculous, and prior to the operations the bacilli were incapsulated. The sore became worse, the plaintiff sought medical advice, and eventually it was decided that amputation was necessary. The defendant's case was that as soon as he had seen the plaintiff's knee he recognised that sooner or later the tuberculosis in the joint would necessitate amputation and he urged Mr. Thomas to see a surgeon. He consented, however, to see him from time to time because he hoped the swelling would be reduced by radiant heat baths. There was no truth in the allegation that he had used force; he merely examined the joint and the anæsthetic was administered because rigidity was essential. A number of witnesses were called on both sides and the hearing of the action occupied three days. In summing up, Mr. Justice Darling said that if at the first examination (assuming it were an examination) the defendant had really told Mr. Thomas the case was hopeless why did he put him to the expense of another five guineas for a second examination? The crux of the case was what was done on the two days in question. The jury returned a verdict for the plaintiff for £21, and judgment was entered accordingly, with costs.

### UNSUCCESSFUL CLAIM AGAINST A DENTIST.

At Lewes Assizes, before Mr. Justice Bucknill and a special jury, Winifred Goble claimed damages from Mr. A. C. Gwatkin, L.D.S., an honorary dental surgeon of the Brighton, Hove, and Preston Dental Hospital, for alleged negligence in the extraction of a tooth at the hospital. Plaintiff's case was that after the extraction of the tooth she was unable to move her jaw. Four days later she again went to the hospital and another dental surgeon extracted two teeth. On the advice of Mr. A. G. Whitehorne-Cole, M.R.C.S., L.R.C.P., she underwent an operation at St. Mary's Hospital, Paddington, for dislocation of the jaw. Mr. Barker, house-surgeon, and Mr. J. E. Lane, F.R.C.S., senior surgeon at St. Mary's Hospital, said they considered an operation essential. The defendant denied dislocating the jaw, and Mr. Stoner, who attended plaintiff on her second visit to the dental hospital, said that if her jaw had been dislocated he must have noticed it.

Dr. E. C. Maguire, who administererd the anæsthetic when the teeth were extracted, said he saw no dislocation. Mrs. Ruth Parsons, matron-nurse at the dental hospital, said the plaintiff made no complaint, and she did not see any sign of dislocation. Dr. Pocock said that about a fortnight after the first tooth was extracted the contraction of the muscles of the left side of the face became very marked. It was a neurotic case. Mr. A. H. Buck, F.R.C.S., also said it was a neurotic case. The jury returned a verdict for the defendant.

